# SPOCK1 promotes the progression of breast cancer by modulating cancer-associated fibroblasts and exerts a synergistic effect with ANXA2

**DOI:** 10.3389/fonc.2025.1619171

**Published:** 2025-08-05

**Authors:** Yuan Jie, Xin Fei, Meng Fan

**Affiliations:** ^1^ Department of Orthopedics, Tianjin First Central Hospital, School of Medicine, Nankai University, Tianjin, China; ^2^ Department of Oncology, Haihe Hospital, Tianjin University, Tianjin, China

**Keywords:** SPOCK1, breast cancer, AnxA2, tumor microenvironment, cancer-associated fibroblasts

## Abstract

**Background:**

SPOCK1, a matricellular glycoprotein, has been implicated in tumor progression, metastasis, and the tumor immune microenvironment, yet its specific roles in breast cancer (BRCA) remain unclear. This study aimed to systematically explore the expression pattern, prognostic significance, mutation landscape, immune association, and spatial localization of SPOCK1 in breast cancer through integrated multi-omics analyses.

**Methods:**

Transcriptomic, genomic, and clinical data from The Cancer Genome Atlas (TCGA) and Gene Expression Omnibus (GEO) were utilized. Bulk RNA sequencing and single-cell RNA sequencing (scRNA-seq) analyses were conducted, including functional enrichment, immune infiltration assessments, mutation profiling, and transcription factor activity analysis. Multiplex immunohistochemistry (mIHC) was performed to validate the spatial distribution of SPOCK1+ cancer-associated fibroblasts (CAFs) within the tumor microenvironment. Statistical analyses were performed using R and GraphPad Prism.

**Results:**

SPOCK1 was broadly overexpressed in multiple cancer types and significantly associated with poor prognosis in BRCA. High SPOCK1 expression correlated with immune checkpoint activation, enhanced immune infiltration, and enriched metastasis-related pathways such as epithelial–mesenchymal transition (EMT) and TGF-β signaling. Single-cell analysis identified CAFs as the primary cell population expressing SPOCK1, with spatial mIHC confirming their close proximity to tumor cells. Furthermore, SPOCK1-high CAFs exhibited stronger intercellular communications with malignant cells via collagen, fibronectin, and IGFBP signaling pathways, alongside distinct transcription factor and metabolic profiles. In breast cancer CAF cell lines with knockdown of ANXA2 we found that the expression of both SPOCK1 and IGF1 was reduced.

**Conclusion:**

SPOCK1 serves as a critical regulator of breast cancer progression, influencing tumor metastasis and reshaping the immune microenvironment via CAF-mediated mechanisms. These findings suggest that targeting SPOCK1+ CAFs could offer new therapeutic opportunities for breast cancer treatment.

## 1 Introduction

Breast cancer is one of the most frequently diagnosed malignancies among women, with distant metastasis—particularly bone metastasis—being a major contributor to poor prognosis and mortality. The bone is the most common site of distant metastasis in breast cancer, with approximately 70–80% of advanced-stage patients developing bone metastases. These patients often suffer from complications such as pathological fractures, pain, hypercalcemia, and nerve compression, all of which are associated with significantly worse outcomes (1). The development of bone metastases is not solely dependent on the intrinsic invasiveness of tumor cells but is also heavily influenced by the tumor microenvironment (TME). Numerous studies have demonstrated that the tumor microenvironment plays a critical role in the initiation and progression of breast cancer. A study by G. Konjević et al. revealed that the activity of natural killer (NK) cells in breast cancer patients significantly decreases with advancing clinical stage, indicating that NK cell dysfunction is associated with tumor progression (2).

In recent years, numerous studies have demonstrated that cancer-associated fibroblasts (CAFs) play a central role in promoting the metastasis of cancer cells to bone tissue (3). As one of the predominant stromal cell types in the TME, CAFs contribute to tumor progression through various mechanisms, including the secretion of cytokines, remodeling of the extracellular matrix (ECM), and induction of epithelial–mesenchymal transition (EMT) in cancer cells (4).

Sparc/osteonectin, cwcv and kazal-like domains proteoglycan 1 (SPOCK1), also known as TIC1, SPOCK, or TESTICAN, is a member of the testican family of multidomain proteoglycans and is implicated in cell proliferation and metastasis (5). SPOCK1 has been reported in various cancers, where it is involved in promoting cell proliferation, angiogenesis, and EMT (6, 7). Recent studies have shown that SPOCK1 is overexpressed in pancreatic and ovarian cancers (6, 8). Furthermore, it has been reported to facilitate invasion and metastasis in gastric cancer and glioma (9, 10), and is associated with poor prognosis in urothelial carcinoma (11).

To date, the limited research on SPOCK1 in breast cancer has shown that it enhances cell proliferation, cell cycle progression, and EMT through activation of the AKT/mTOR signaling pathway, and that these effects can be reversed by either inhibiting AKT/mTOR signaling or depleting SIX1 (12). However, the underlying mechanisms by which SPOCK1 contributes to breast cancer progression and metastasis—particularly its functional role within the tumor microenvironment—remain largely unclear. This study aims to further investigate the role of SPOCK1 in breast cancer, focusing specifically on its function in cancer-associated fibroblasts.

## 2 Materials and methods

### 2.1 Data acquisition

Breast cancer (BRCA) transcriptomic data were obtained from The Cancer Genome Atlas (TCGA) via the UCSC Xena platform (http://xenabrowser.net/hub). All single-cell analyses were based on datasets downloaded from the Gene Expression Omnibus (GEO) database, hosted by the National Center for Biotechnology Information (NCBI) (http://www.ncbi.nlm.nih.gov/geo/). Single-cell RNA sequencing (scRNA-seq) data of breast cancer and adjacent normal breast tissues were retrieved from GEO accession GSE243526, which contains 16 individual samples. To eliminate batch effects, the data were integrated using standard single-cell analytical workflows. Since all data used in this study were obtained from publicly available databases, no institutional ethical approval was required.

### 2.2 scRNA-seq data processing and dimensionality reduction

The GSE243526 scRNA-seq dataset was downloaded from the GEO database (https://www.ncbi.nlm.nih.gov/geo/). The data were processed using the Seurat R package (version 5.1.0) (13). Mitochondrial gene content was calculated using the PercentageFeatureSet function. Cells expressing fewer than 250 genes, with mitochondrial content exceeding 10%, with fewer than 500 UMI counts, or with log10GenesPerUMI values lower than 0.8 were excluded. After quality control, a total of 102,221 cells from 16 samples were retained. The integrated Seurat object was normalized and principal component analysis (PCA) was conducted, with the top 30 principal components selected for clustering. Uniform Manifold Approximation and Projection (UMAP) was used for dimensionality reduction. To correct for batch effects, the Harmony algorithm was applied. Clustering was performed using the FindClusters function in Seurat, and cluster resolution was optimized using the “clustree” R package. The final clustering resolution was set to 0.2 (14).

### 2.3 Cell type marker selection

Cell-type-specific markers for breast cancer were compiled from previous studies (15, 16) and the CellMarker 2.0 database. The following markers were used: Epithelial cells: EPCAM, KRT18, KRT19, CLDN4; Natural killer T cells: NKG7, KLRD1, GNLY, CD3D, CD3E, CD2; B cells: MS4A1, CD79A, CD79B, IGHG1; Macrophages: SSP1, CD68, IL1B, CD163; Cancer-associated fibroblasts (CAFs): COL1A2, COL3A1, DCN, THY1; Endothelial cells: PECAM1, VWF, CDH5, PLVAP; Mast cells: CPA3, GATA2, MS4A2, TPSB2.

### 2.4 Functional enrichment analysis

Differentially expressed genes (DEGs) were subjected to Gene Ontology (GO) enrichment analysis using the R packages “clusterProfiler” and “enrichplot,” with statistical significance set at P < 0.05. Gene sets with P < 0.05 and a false discovery rate (FDR) q-value < 0.05 were considered significantly enriched. GO and Kyoto Encyclopedia of Genes and Genomes (KEGG) pathway analyses were performed for each cell cluster, integrating marker gene information to elucidate the potential roles of SPOCK1 in different cellular populations (17).

### 2.5 Immune cell infiltration, immune function, and immune checkpoint analysis

Single-sample gene set enrichment analysis (ssGSEA) was performed using the GSVA package to obtain Hallmark gene set scores and assess the immune landscape based on bulk RNA-seq data. The relative infiltration levels of 28 immune cell types were quantified in breast cancer samples. These immune cell scores showed significant variability. Spearman correlation analysis was then used to correlate immune infiltration scores with key gene expression levels, and results were visualized using the ggplot2 package. Immune checkpoint molecules analyzed included LAG3, PDL1, PD1, TIGIT, CTLA4, VISTA, BTLA, and TIM3 (14).

### 2.6 Somatic mutation data analysis

Somatic single-nucleotide variant (SNV) data, processed using the Mutect pipeline, were visualized and analyzed using the “maftools” R package (version 2.4.05) (18).

### 2.7 Multiplex immunohistochemistry

Immunohistochemistry (IHC) was performed to assess the expression of SPOCK1+ CAFs in breast cancer tissues and their spatial association with tumor cells. Tissue microarrays (TMAs) underwent antigen retrieval using EDTA buffer (pH 9) at boiling temperature for 15 minutes, followed by endogenous peroxidase inactivation with 3% hydrogen peroxide. After blocking with 3% BSA for 30 minutes, sections were incubated overnight at 4°C with primary antibodies. The next day, sections were washed with PBS and incubated for 50 minutes at room temperature with HRP-conjugated secondary antibodies, followed by DAB chromogenic detection and hematoxylin counterstaining. Triple IHC staining employed the following antibodies: α-SMA (Mouse, ab5831, Abcam, Hunan, 1:100), GATA3 (mouse, AF20162, Affinity Biosciences, Hunan, 1:100), and SPOCK1 (rabbit, EPR16096, Affinity Biosciences, Shanghai, 1:100) to visualize spatial distribution and potential interactions of these cells within the tumor microenvironment.

### 2.8 Experimental models and patient information

This study protocol was approved by the Ethics Committee of Tianjin First Central Hospital. The Academic Research Office and the Ethics Committee confirmed that the study conformed to the Declaration of Helsinki and other relevant ethical guidelines for human research. All breast cancer tissue samples were obtained from surgically resected T1-stage tumors. Patients had not received any preoperative treatment or had major comorbidities. All participants provided written informed consent, permitting the use of their gender, age, and institutional affiliation for research purposes.

### 2.9 Cell culture and lentiviral infection

Human breast cancer-associated fibroblasts (CAFs) were obtained from Procell Life Science & Technology Co., Ltd. (Wuhan, China; CP-H172) and cultured in CM-H172 medium. All culture media were supplemented with 1% penicillin/streptomycin and 10% fetal bovine serum (FBS, Gibco, USA). Cells were maintained in a humidified atmosphere at 37°C with 5% CO_2_.

A human siRNA sequence targeting ANXA2 (siANXA2-1, 5’-TAGGTCTGAATTCAAGAGAAA-3’) was cloned into the pLKO.1-puro vector to achieve endogenous knockdown of ANXA2. For lentivirus production, 293T cells were co-transfected with the lentiviral vector and packaging plasmids using Lipofectamine 2000 (Invitrogen, Shanghai, China) according to the manufacturer’s instructions. The culture supernatants were collected at 48 hours and 72 hours post-transfection, pooled, and filtered. Subsequently, the indicated cell lines were infected with the harvested lentiviruses.

### 2.10 Western blotting

Proteins were separated from cell lines Human breast cancer-associated fibroblasts utilizing radio-immunoprecipitation assay (RIPA) lysis buffer. A bicinchoninic acid (BCA) protein kit (Beyotime Biotechnology, Shanghai, China) was then utilized for protein quantifications. Before being transferred to polyvinylidene fluoride (PVDF) membranes, materials were electrophoresed on a 10% SDS polyacrylamide gel (SDS-PAGE) (Merck Millipore, Billerica, MA, USA). Blocking in 5% nonfat milk and tris-buffered saline buffer (TBST), then membranes were treated with IGF-1 (ab182408, Abcam, Shanghai, China), ANXA2(ab185957, Abcam, Shanghai, China), SPOCK1 (ab229935, Abcam, Shanghai, China) and β-actin (ab8227, Abcam) overnight at 4°C, after that incubated with HRP-conjugated secondary antibody (Sangon, Shanghai, China) for 1 h at 37°C. Enhanced chemiluminescence revealed protein bands (ECL; Beyotime) followed by analysis utilizing ImageJ software (19).

### 2.11 Statistical analysis

Wilcoxon rank-sum tests were used for comparisons between two groups, while one-way analysis of variance (ANOVA) was used for comparisons involving three or more groups. All statistical analyses were conducted using RStudio (version 4.2.2) and GraphPad Prism (version 8.0). Statistical significance was defined as P < 0.05.

## 3 Results

### 3.1 Pan-cancer analysis of SPOCK1 expression

**Figure 1:**
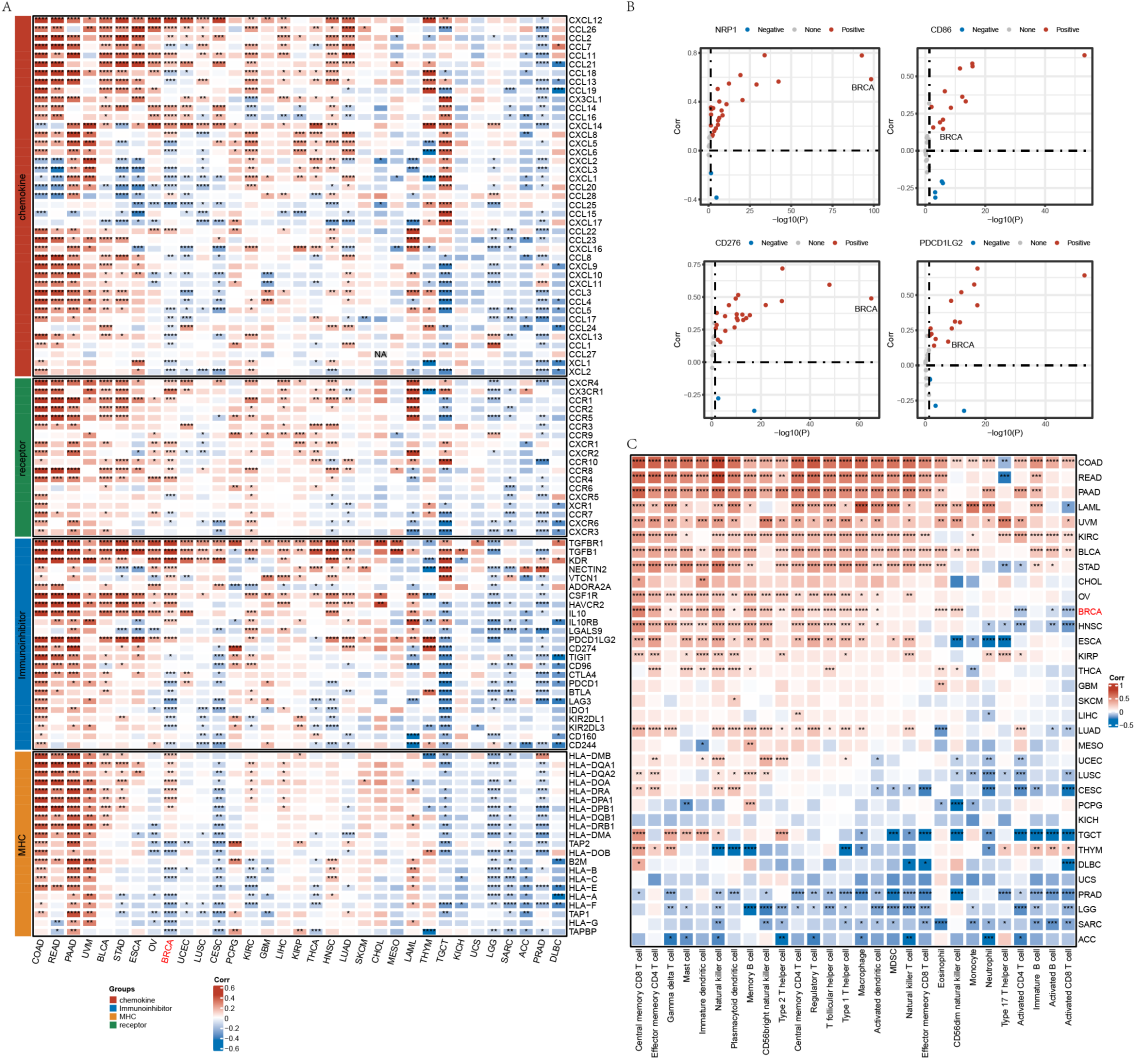
**(A)** Correlation analysis of gene SPOCK1 with characterized genes in pan-cancer; **(B)** Correlation of some genes with immune genes in pan-cancer; **(C)** Correlation of gene SPOCK1 with immune cell score in pan-cancer. *P < 0.05; **P < 0.01; ***P < 0.001; ****P < 0.0001.

We downloaded RNA-Seq data, single nucleotide variant (SNV) mutation data, and clinical follow-up information across 33 cancer types from The Cancer Genome Atlas (TCGA) database (https://portal.gdc.cancer.gov/). Additionally, we obtained RNA-Seq and clinical data from 32 other cancer types. Immune-related gene sets, including chemokines, immunoinhibitors, major histocompatibility complex (MHC), and receptors, were retrieved from the TISIDB database (20), and their correlation with SPOCK1 was analyzed across cancers (**Figure 1A**). Furthermore, we assessed the correlation between SPOCK1 and immune checkpoint genes (e.g., PD-L1 and CD8), previously reported to define immunologically favorable subtypes (21). Our results showed that SPOCK1 expression is significantly positively correlated with these immune genes in most cancer types (**Figure 1B**).

Using the single-sample gene set enrichment analysis (ssGSEA) method and immune cell marker genes, we computed immune cell infiltration scores and examined their correlation with SPOCK1 expression. We observed significant positive correlations in breast invasive carcinoma (BRCA), colon adenocarcinoma (COAD), pancreatic adenocarcinoma (PAAD), and kidney renal clear cell carcinoma (KIRC), and significant negative correlations in sarcoma (SARC), prostate adenocarcinoma (PRAD), brain lower grade glioma (LGG), and adrenocortical carcinoma (ACC) (**Figure 1C**).

### 3.2 Association between SPOCK1 and prognosis in breast cancer

**Figure 2:**
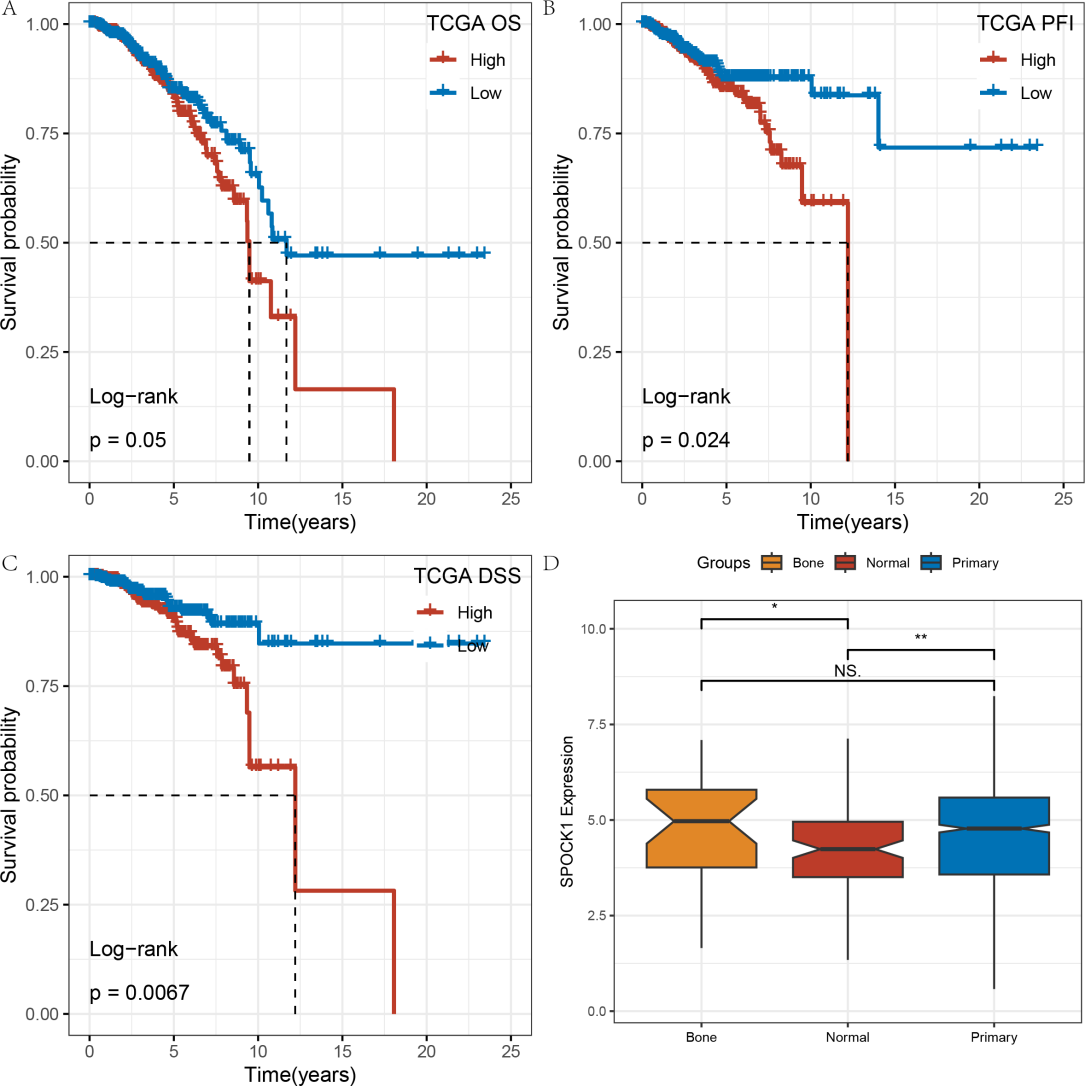
**(A–C)** KM curves of gene SPOCK1 at different times and states on the TCGA dataset; **(D)** expression differences of gene SPOCK1 in different subgroups. (Normal refers to normal samples, Primary refers to primary tumor samples, and Bone refers to tumor samples that have undergone bone metastasis). *P < 0.05; **P < 0.01.

In the TCGA-BRCA dataset, Kaplan–Meier (KM) survival curves for overall survival (OS) demonstrated that high SPOCK1 expression is associated with poorer prognosis. Significant differences were also observed in progression-free interval (PFI) and disease-specific survival (DSS) analyses (**Figures 2A–C**).

Additionally, we compared SPOCK1 expression levels among normal tissues, primary breast tumors, and bone metastatic samples. SPOCK1 expression was higher in both primary tumors and bone metastases compared to normal tissues. Although no statistically significant difference was observed between bone metastatic and primary tumor groups—possibly due to the small number of bone metastasis samples—SPOCK1 expression tended to be higher in bone metastases (**Figure 2D**).

### 3.3 Mutation analysis based on SPOCK1 expression subtypes

**Figure 3:**
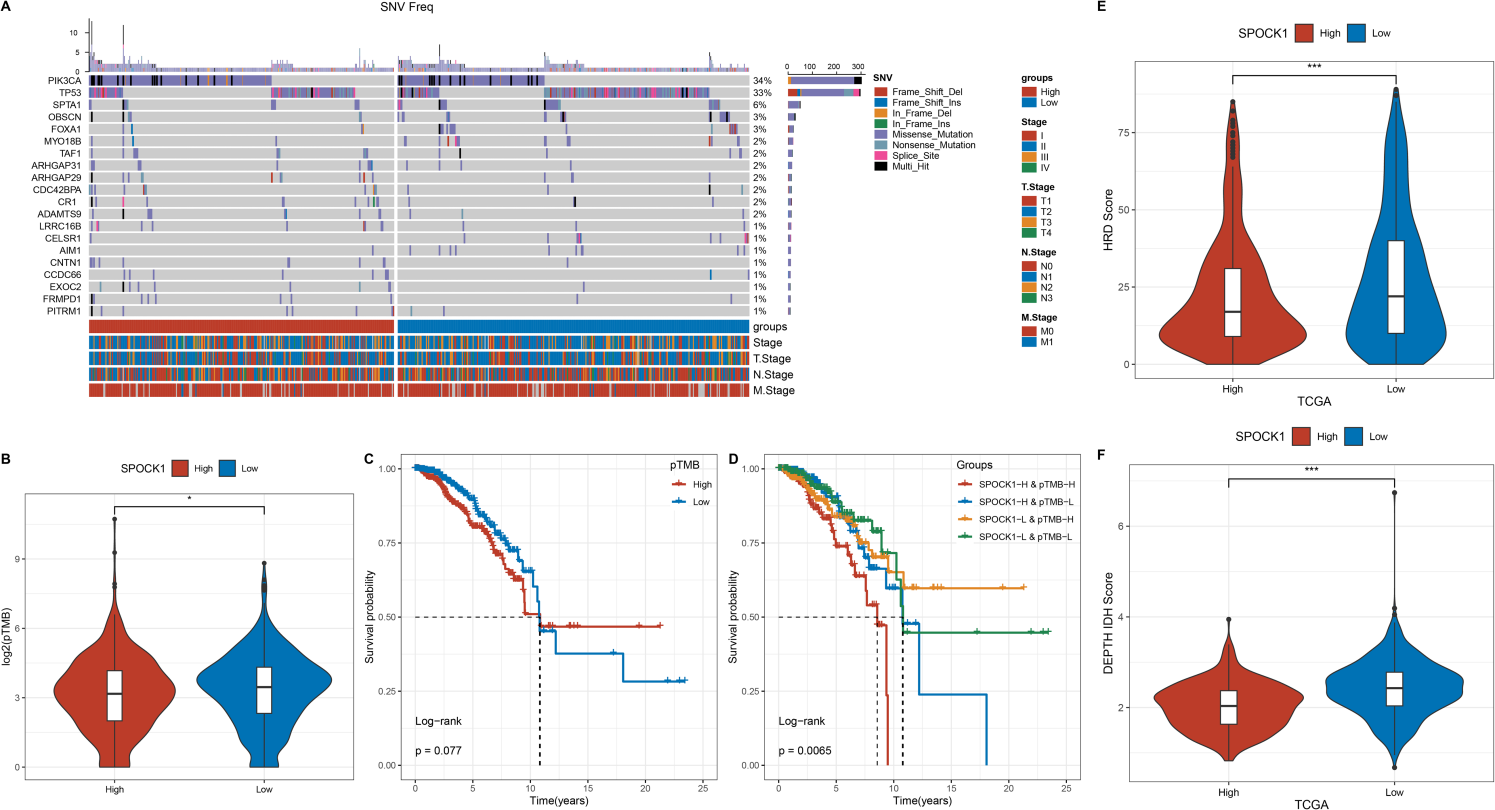
**(A)** Mutation frequency distribution of the top 20 tumor driver genes with differential mutation frequencies in molecular subtypes; **(B)** Comparison of TMB on molecular subtypes; **(C)** Survival curves of some mutated genes; **(D)** Density maps of immune checkpoint genes; **(E)** Difference of HRD Score in high and low expression groups; **(F)** Difference of intratumor in high and low expression groups. heterogeneity difference. *P < 0.05; ***P < 0.001.

Samples were divided into high and low SPOCK1 expression groups based on the median value. Genes with at least three mutations were retained, and chi-square tests were used to identify differentially mutated genes between the two groups (p < 0.05). A total of 83 genes were found to be significantly differentially mutated. Among the top mutated genes, the mutation frequency of PIK3CA was lower in the high expression group, while TP53, SPTA1, OR, and FOXA1 mutations were more frequent in the low expression group (**Figure 3A**).

Persistent tumor mutation burden (pTMB) data were obtained from a previous study (Persistent mutation burden drives sustained anti-tumor immune responses), and its levels were compared between the two groups (**Figure 3B**). KM survival analysis showed that high pTMB was associated with worse prognosis, and this trend was also significant when combined with SPOCK1 expression grouping (**Figures 3C, D**).

Homologous recombination deficiency (HRD) scores were retrieved from a TCGA-wide analysis of DNA damage repair deficiency (22). HRD scores were significantly higher in the low SPOCK1 expression group (**Figure 3E**). Similarly, intratumor heterogeneity (ITH) scores were evaluated using the DEPTH package (23), and ITH followed the same trend as HRD (**Figure 3F**).

### 3.4 Correlation between SPOCK1 and tumor metastasis

**Figure 4:**
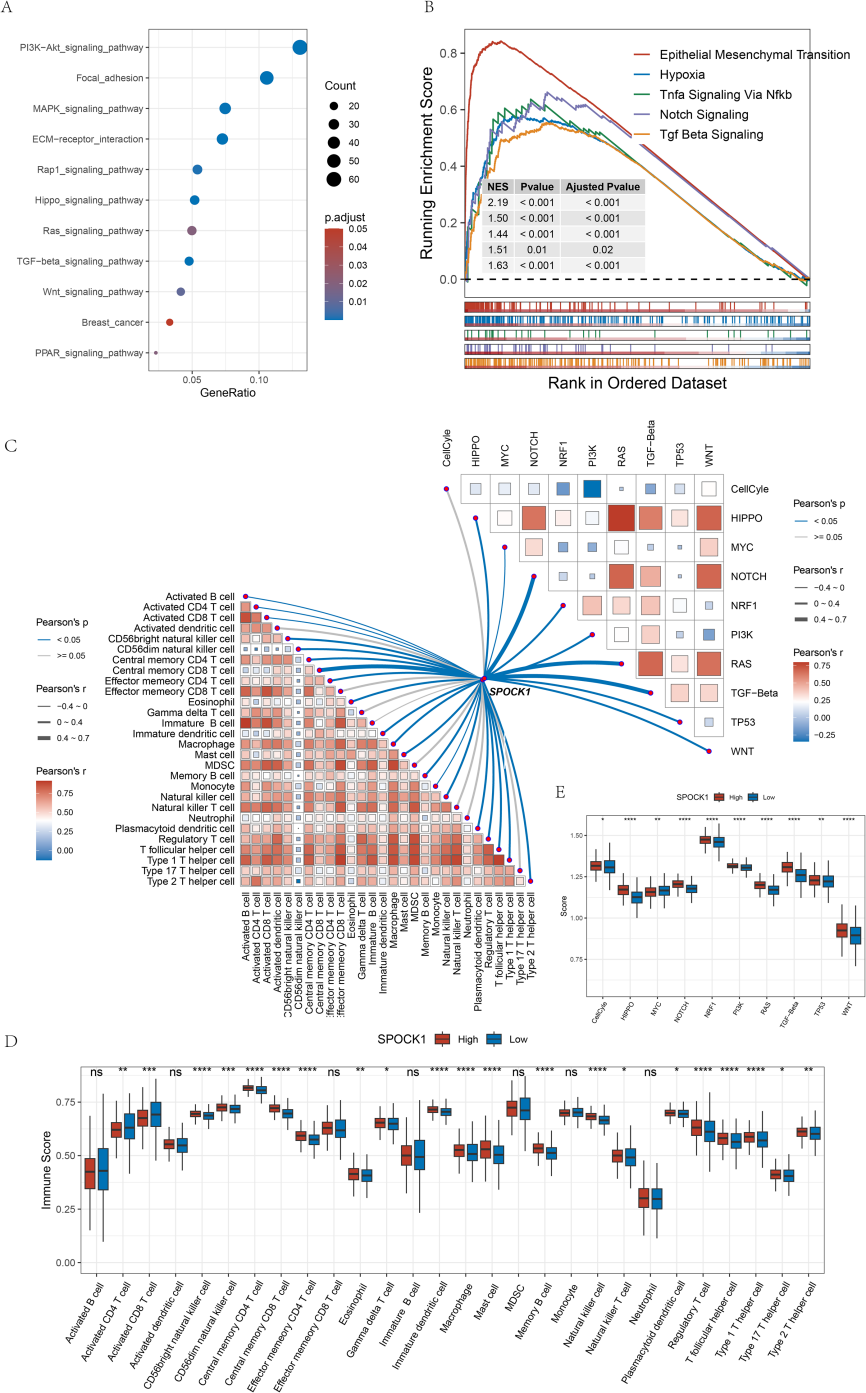
**(A)** KEGG pathway enrichment analysis of up-regulated genes in the high-expression subgroup; **(B)** some pathways significantly enriched to in the high-expression subgroup; **(C)** correlation analysis of the gene SPOCK1 with oncogenic pathway scores as well as immune cell scores in the TCGA dataset are shown; **(D)** difference analysis of immune cell scores in the subgroups; **(E)** difference analysis of 10 pathway scores in the subgroups. *P < 0.05; **P < 0.01; ***P < 0.001; ****P < 0.0001.

We identified 1,040 differentially expressed genes (DEGs) between SPOCK1 high and low expression groups in TCGA-BRCA, using |fold change| > 1.5 and false discovery rate (FDR) < 0.05 as thresholds, including 1,027 upregulated and 13 downregulated genes. Kyoto Encyclopedia of Genes and Genomes (KEGG) enrichment analysis of the upregulated genes revealed significant enrichment in metastasis-related pathways, including Wnt signaling pathway, as well as ECM-receptor interaction, PI3K-Akt signaling, TGF-beta signaling, Rap1 signaling, Hippo signaling, breast cancer pathway, MAPK signaling, focal adhesion, Ras signaling, and PPAR signaling pathways (**Figure 4A**).

Gene Set Enrichment Analysis (GSEA) further indicated that genes upregulated in the SPOCK1-high group were enriched in epithelial–mesenchymal transition (EMT), Notch signaling, hypoxia, TNF-α signaling via NF-κB, and TGF-β signaling pathways (**Figure 4B**).

### 3.5 Association of SPOCK1 with pathways and the tumor immune microenvironment

Correlation analysis between SPOCK1 and 28 immune cell types revealed mostly significant positive correlations (**Figure 4C**, lower left). Moreover, 19 immune cell types showed significantly higher scores in the high SPOCK1 expression group (**Figure 4D**).

We also analyzed 10 oncogenic signaling pathways retrieved from a previous TCGA study (24) using ssGSEA and observed strong positive correlations between SPOCK1 and most pathways (**Figure 4C**, upper right), especially those associated with metastasis such as Notch and Wnt pathways. Except for the MYC pathway, scores for the remaining pathways were significantly higher in the SPOCK1-high group (**Figure 4E**).

### 3.6 Single-cell clustering and cell type annotation analysis

To investigate the single-cell heterogeneity landscape of breast cancer, we utilized a published dataset (GSE243526), which includes 12 breast tumor and 4 adjacent normal samples. A total of 102,221 cells were analyzed. Dimensionality reduction was performed, and batch effects were corrected using the “Harmony” algorithm. Clustering was based on the top 30 principal components with a resolution of 0.2, resulting in 15 clusters (**Supplementary Figure S1A**).

**Figure 5:**
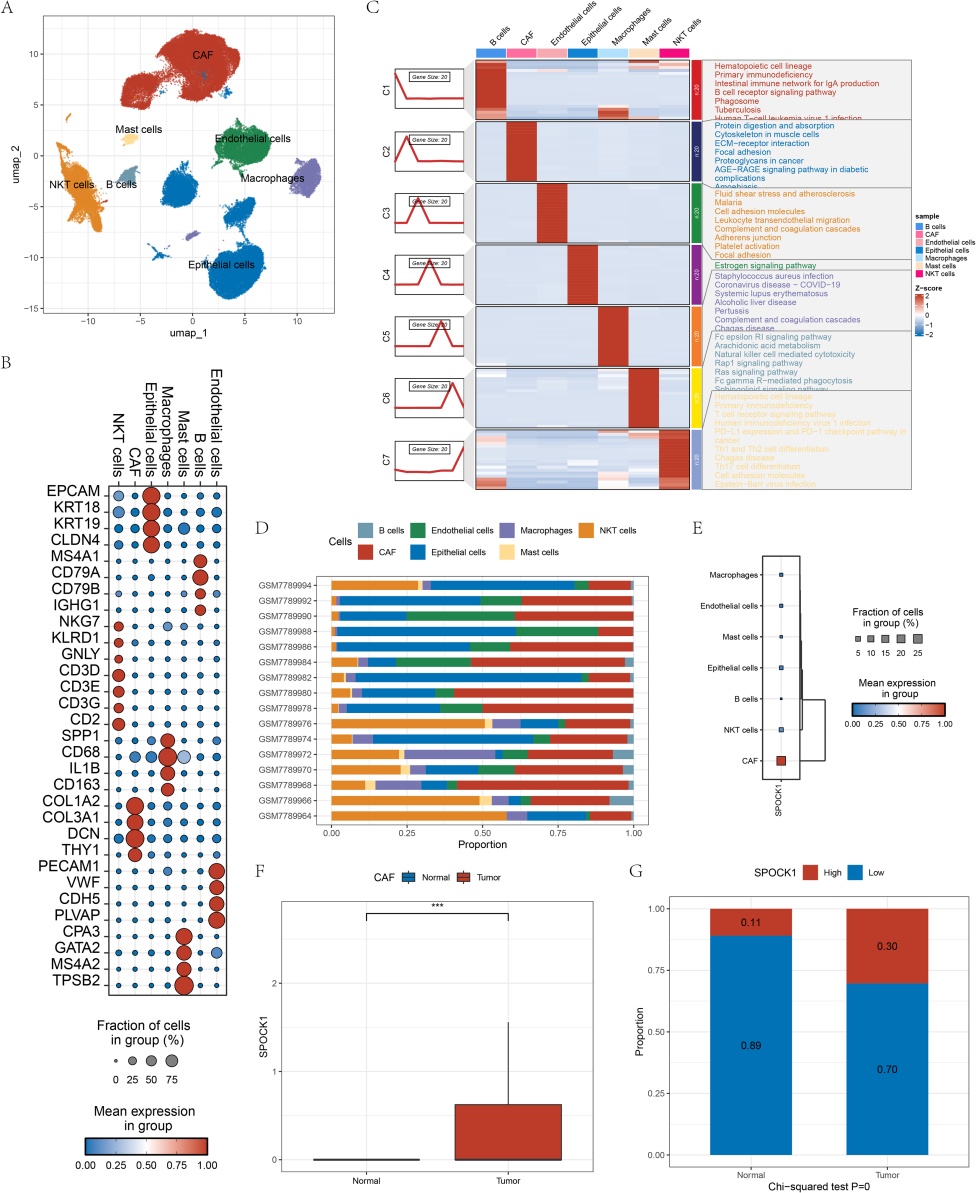
**(A)** Cellular annotation UMAP plot after single-cell dimensionality reduction clustering; **(B)** Cellular maker gene expression display plot in cells; **(C)** KEGG functional enrichment scores of different maker genes in different cells; **(D)** Plot of the proportion of cells in different samples; **(E)** Gene expression of the gene SPOCK1 in the cells; **(F)** Gene SPOCK1 in the CAF cells expression differences in normal and tumor samples; **(G)** differences in the distribution of high and low SPOCK1 groupings in CAF cells in normal and tumor samples. ***P < 0.001.

Using marker genes obtained from the CellMarker 2.0 database, we annotated seven major cell types: epithelial cells (EPCAM, KRT18, KRT19, CLDN4), NKT cells (NKG7, KLRD1, GNLY, CD3D, CD3E, CD2), B cells (MS4A1, CD79A, CD79B, IGHG1), macrophages (SSP1, CD68, IL1B, CD163), cancer-associated fibroblasts (CAFs; COL1A2, COL3A1, DCN, THY1), endothelial cells (PECAM1, VWF, CDH5, PLVAP), and mast cells (CPA3, GATA2, MS4A2, TPSB2) (**Figure 5B**). Clusters were visualized by UMAP (**Figure 5A**).

The accuracy of annotations was validated using the FindAllMarkers function, with filtered marker genes (fold change > 2, FDR < 0.05), followed by functional enrichment analysis (**Figure 5C**). All cell types were observed across all samples, supporting robustness of classification (**Figure 5D**). SPOCK1 expression was highest in CAFs, a cell type known to play a vital role in tumor development and metastasis (**Figure 5E**). Furthermore, CAFs in tumor samples showed significantly higher SPOCK1 expression than those in normal samples (**Figure 5F**). CAFs were stratified into SPOCK1-high (expression > 0) and SPOCK1-low (expression = 0) groups. In tumor samples, 30% of CAFs belonged to the high expression group, versus only 11% in the low expression group (**Figure 5G**).

### 3.7 Cell–cell communication analysis

We identified seven major cell types in BRCA samples. Given the high SPOCK1 expression in CAFs, and its elevated levels in tumor versus normal tissues, CAFs were stratified into SPOCK1-high (CAF+SPOCK1) and SPOCK1-low (CAF−SPOCK1) groups. In breast cancer research, Vladimir Jurisic has demonstrated that cytokines, as key signaling molecules produced by both cancer cells and immune cells, play a critical role in modulating the tumor microenvironment, mediating immune responses, and either promoting or inhibiting tumor progression (25). To assess the interaction between these subtypes and other cells, we conducted a cell–cell communication analysis.

**Figure 6:**
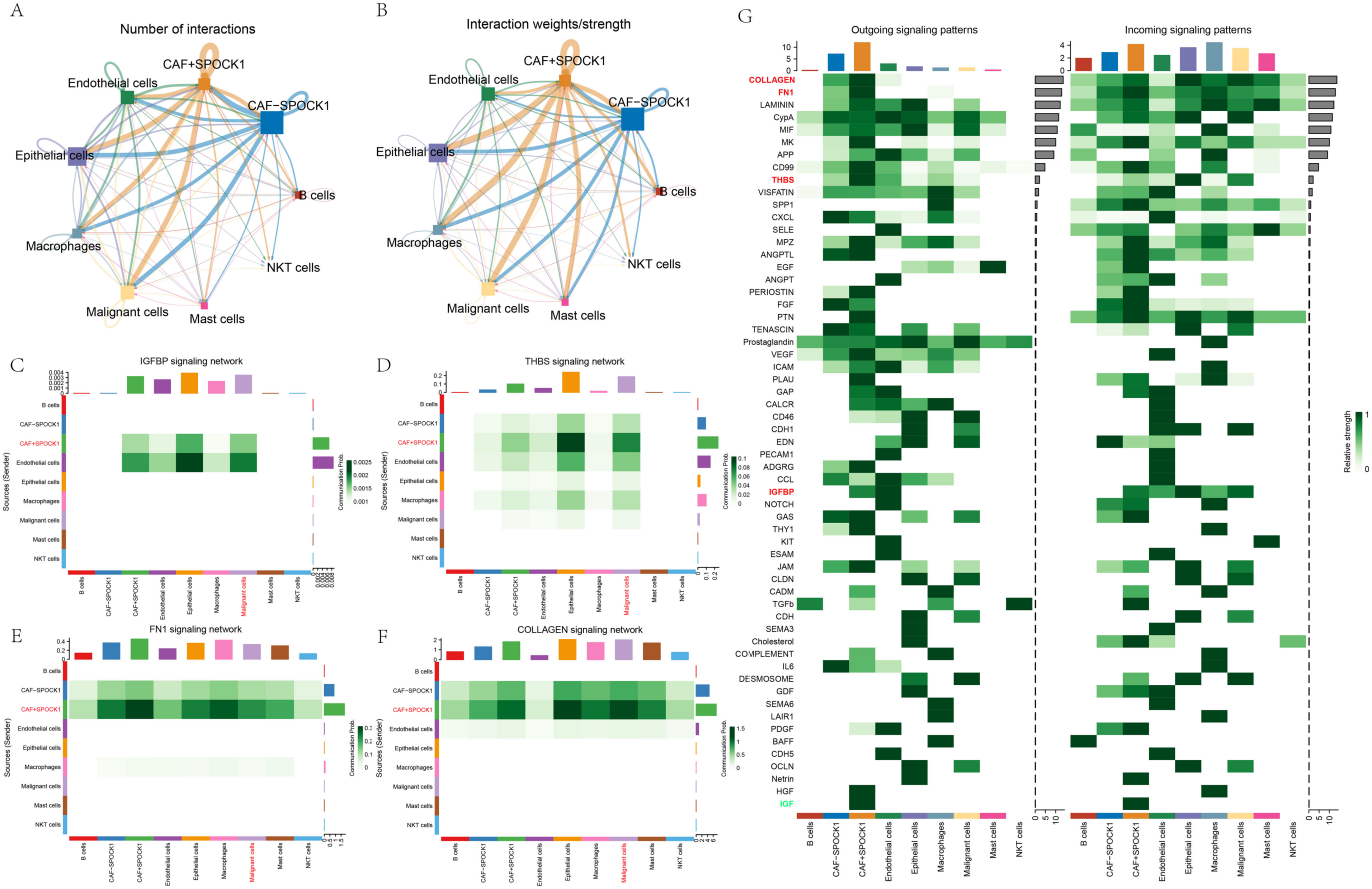
**(A)** Direction of communication as well as the number of exchanges between cells; **(B)** Direction of communication as well as intensity of exchanges between cells; **(C-F)** Communication diagrams of OLLAGEN signaling, FN1 signaling, THBS signaling and IGFBP signaling between cells; **(G)** Identification of different cell populations of the output or input signaling that contributes the most to the signaling.

Results revealed complex interactions among cell types, with stronger communication weights observed between CAF+SPOCK1 and malignant epithelial cells compared to CAF−SPOCK1 (**Figures 6A, B**). Key signaling pathways mediating this interaction included collagen, fibronectin 1 (FN1), thrombospondin (THBS), and insulin-like growth factor-binding proteins (IGFBPs) (**Figures 6C–G**).

Additionally, malignant epithelial cells were stratified by HER2 status into Malignant−HER2 and Malignant+HER2. In HER2-positive malignant cells, the four aforementioned signaling pathways also dominated communication with CAF+SPOCK1 cells (**Supplementary Figure S1B**).

### 3.8 Transcription factor and metabolic pathway analysis

**Figure 7:**
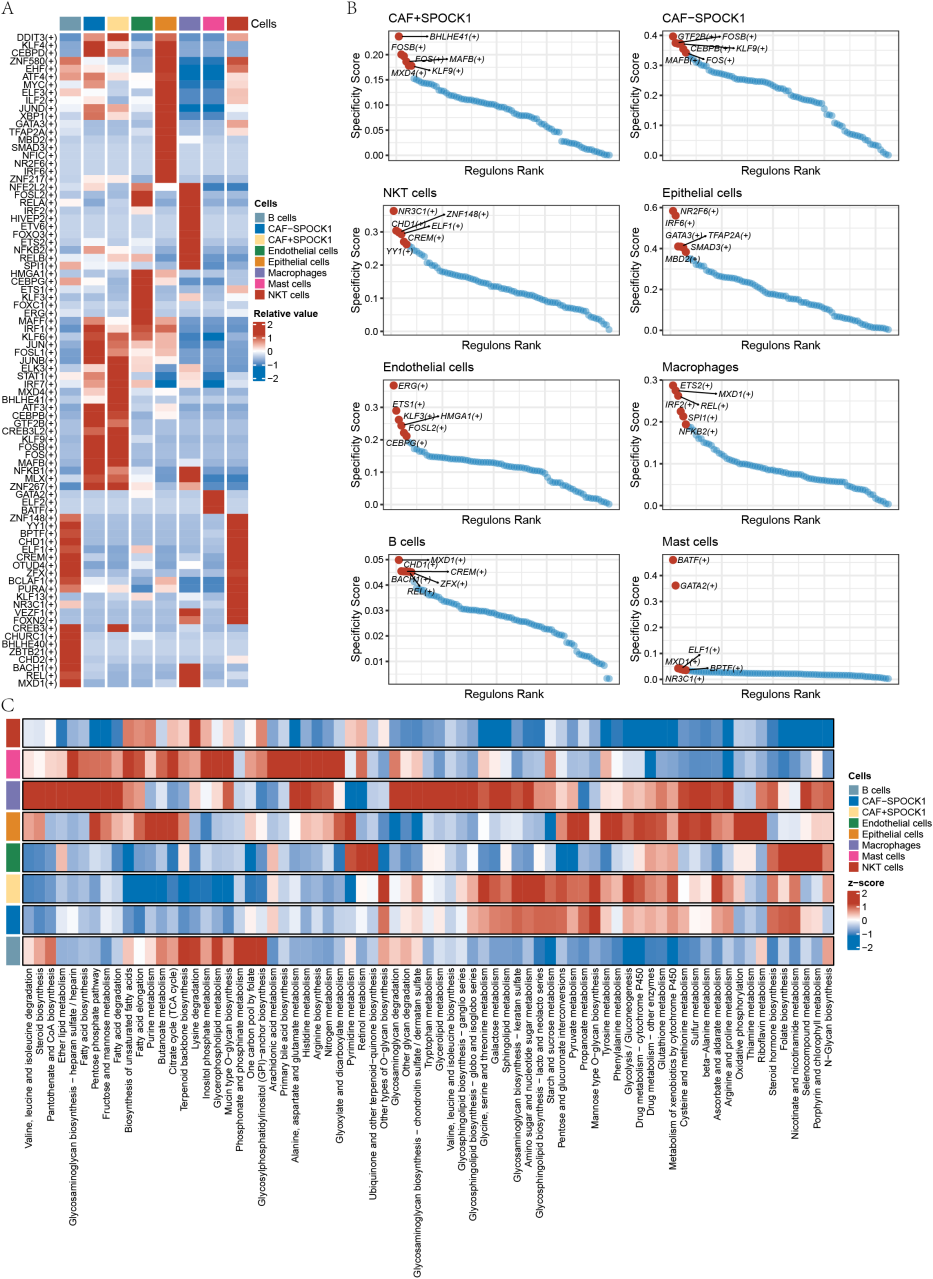
**(A)** Heatmap display of relevant transcription factors in different cells; **(B)** Display of transcription factors with the highest Specificity Score in different cells; **(C)** Heatmap analysis of the scoring of metabolite pathways in cell subtypes.

Transcription factors (TFs) regulate gene expression by binding to DNA at specific motifs and are essential in processes such as immunity and development. We used the SCENIC (Single-Cell rEgulatory Network Inference and Clustering) framework to infer TF activity from single-cell RNA-sequencing data. The heatmap in **Figure 7A** illustrates TF activity across cell types. For each cell type, we selected the six most relevant TFs, highlighting BHLHE41 and MXD4 as highly correlated in CAF+SPOCK1 cells (**Figure 7B**).

Cell-type-specific metabolic differences were also analyzed using scMetabolism. Metabolic pathways significantly associated with CAF+SPOCK1 included sphingolipid metabolism, glycolysis/gluconeogenesis, metabolism of xenobiotics by cytochrome P450, and ascorbate and aldarate metabolism (**Figure 7C**).

### 3.9 Spatial transcriptomics reveals the relationship between CAF+SPOCK1 and malignant epithelial cells

**Figure 8:**
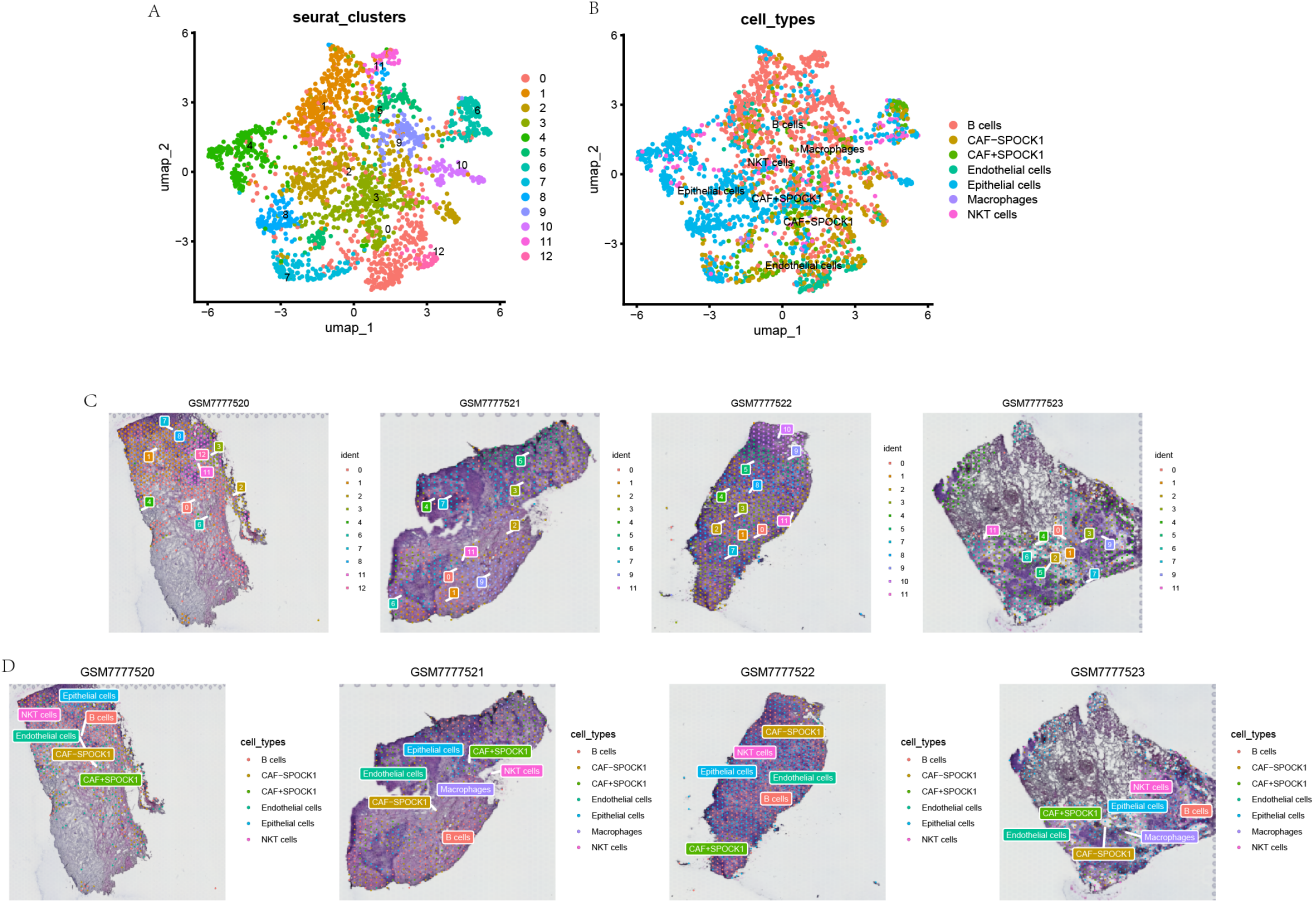
Spatial transcriptomics images of Breast cancer tumor cells and SPOCK1+ CAF cells. **(A, B)** Annotation information from single-cell RNA sequencing was projected onto spatial transcriptomics data, enabling the localization of specific cell types within the tissue context. **(C, D)** Spatial distribution patterns of distinct cell subpopulations were visualized, highlighting their localization and potential spatial interactions within the tumor microenvironment.

To further validate the above findings, we analyzed breast cancer spatial transcriptomics (ST) data to investigate the spatial distribution of cell types. Using SingleR, cell-type-specific scores were computed, revealing co-enrichment of CAF+SPOCK1 and malignant epithelial cells in spatially adjacent tumor regions (**Figure 8**).

**Figure 9:**
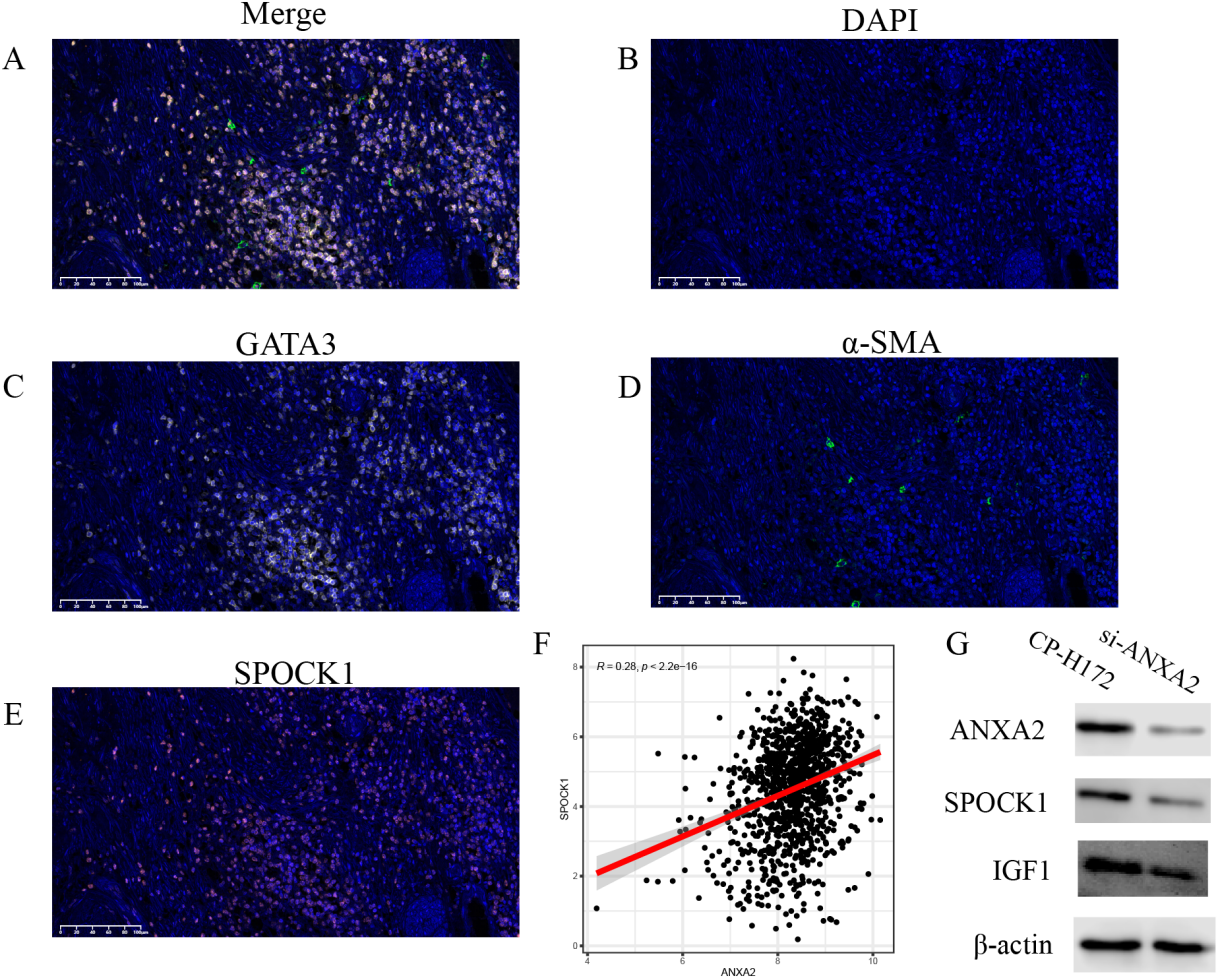
**(A)**, mIHC showing the localization of SPOCK1+ CAF cells and tumor cells in breast cancer tissues; **(B-E)**, mIHC plots showing the marker proteins; **(F)**, positive correlation trend between ANXA2 and SPOCK1 in TCGA; **(G)**, knockdown of ANXA2 from human breast cancer fibroblasts (CP-H172) resulted in reduction of both SPOCK and IGF1.

Multiplex immunohistochemistry (mIHC) further confirmed their spatial co-localization within tumor regions, indicating potential cell–cell communication between CAF+SPOCK1 and malignant epithelial cells (**Figures 9A–E**).

### 3.10 Correlation analysis between SPOCK1 and ANXA2

To further explore the regulatory mechanisms of SPOCK1, we examined its correlation with annexin A2 (ANXA2), finding a significant positive relationship. ANXA2 was also found to be highly expressed in CAFs within the single-cell RNA-sequencing data (**Figure 9F**). A previous study by Yanmei Yi et al. in non-small cell lung cancer (26) demonstrated that CAFs promote ANXA2 expression and phosphorylation via secretion of hepatocyte growth factor (HGF) and insulin-like growth factor 1 (IGF-1), activating c-MET and IGF-1R receptors, thereby enhancing the epithelial–mesenchymal transition (EMT) process. Our cell–cell communication analysis suggested that CAF+SPOCK1 cells interact via the IGF signaling pathway (**Supplementary Figure S1C**). Thus, we hypothesize that CAF+SPOCK1 cells may upregulate ANXA2 expression and phosphorylation through autocrine IGF signaling, further promoting EMT and tumor progression.

Existing evidence indicated that Annexin A2 (ANXA2) is a calcium-dependent phospholipid-binding protein that often functions as a regulatory node in signal transduction, modulating cytoskeletal dynamics, cell migration, adhesion, and exosome function (27). In contrast, SPOCK1 is primarily an extracellular matrix (ECM)-associated protein that predominantly exerts functional roles such as promoting cell invasion, metastasis, and regulating the tumor microenvironment (28). Previous studies have shown that ANXA2 could activate signaling pathways such as PI3K/AKT and TGF-β, which in turn upregulate SPOCK1 expression, thereby promoting epithelial-mesenchymal transition (EMT), invasion, and metastasis (29, 30). Based on these findings, we hypothesized that ANXA2 might act as an upstream regulator of SPOCK1 in breast cancer-associated fibroblasts (CAFs).

To further investigate the interaction between ANXA2 and SPOCK1, we constructed GARS-knockdown breast cancer fibroblasts using ANXA2-specific siRNA. The experimental results confirmed that the expression levels of ANXA2 were significantly decreased at both the mRNA and protein levels. Subsequently, we compared the expression levels of SPOCK1 and IGF-1 between parental breast cancer fibroblasts and ANXA2-knockdown breast cancer fibroblasts. We found that the expression of both SPOCK1 and IGF-1 was reduced in ANXA2-knockdown breast cancer fibroblasts (**Figure 9G**).

## 4 Discussion

This study systematically explored the expression pattern and clinical significance of SPOCK1 (SPARC/osteonectin, CWCV and Kazal-like domains proteoglycan 1) across multiple cancer types, with a particular focus on its role in breast cancer. By integrating bulk RNA sequencing, single-cell transcriptomics, and spatial transcriptomics data, we found that SPOCK1 is generally overexpressed in tumor tissues compared to normal tissues. Notably, high SPOCK1 expression was significantly associated with poor prognosis in breast cancer patients, including reduced overall survival (OS), progression-free interval (PFI), and disease-specific survival (DSS). These findings suggest that SPOCK1 may serve as a potential prognostic biomarker and play a critical role in tumor progression and metastasis.

Under physiological conditions, SPOCK1 is primarily involved in extracellular matrix (ECM) remodeling and intercellular communication. However, in the tumor microenvironment, SPOCK1 appears to be co-opted to promote tumor cell proliferation, migration, and immune evasion. Our pan-cancer analysis revealed that SPOCK1 expression positively correlates with multiple immune-related genes, including chemokines, immunosuppressive molecules, and major histocompatibility complex (MHC) components. Furthermore, SPOCK1 is highly associated with immune checkpoint molecules in several cancers, suggesting that it may influence tumor–immune system interactions by modulating the tumor immune microenvironment, thereby promoting immune tolerance.

Interestingly, mutational landscape analysis demonstrated significant differences in somatic mutation profiles between high and low SPOCK1 expression groups. Specifically, the low SPOCK1 expression group exhibited a higher mutation frequency in TP53 and other tumor suppressor genes, potentially explaining the paradox of increased genomic instability despite lower SPOCK1 expression. Moreover, high SPOCK1 expression was associated with lower tumor mutational burden (TMB) and homologous recombination deficiency (HRD) scores, indicating that SPOCK1 may influence DNA repair capacity and tumor heterogeneity, ultimately contributing to treatment resistance and metastatic potential.

In breast cancer, SPOCK1 exhibited a complex relationship with immune cell infiltration. Single-sample gene set enrichment analysis (ssGSEA) indicated that SPOCK1 expression positively correlated with immune scores in some cancer types but negatively correlated in others. This bidirectional correlation suggests that SPOCK1 may have context-dependent immunomodulatory roles across different tumor immune landscapes. In our study, SPOCK1 expression was significantly elevated in breast cancer tissues, particularly in bone metastases, where it was predominantly expressed in cancer-associated fibroblasts (CAFs). Single-cell transcriptomic analysis further confirmed that SPOCK1 expression was significantly higher in CAFs from tumor tissues than from adjacent normal tissues, implying that SPOCK1 may contribute to breast cancer metastasis, especially bone metastasis, through CAF-mediated mechanisms.

Paulo Roberto Del Valle’s study demonstrated that CAFs are the major stromal component in tumors, secreting various factors to promote tumor cell proliferation, invasion, and metastasis. CAFs from primary breast tumors and lymph nodes exhibited similar transcriptional profiles, which differed markedly from those of bone marrow-derived mesenchymal cells (BMMCs), suggesting that tumor cells may remodel fibroblasts in the primary and lymph node microenvironments through common mechanisms. In contrast, CAFs in bone metastatic tissues showed more active gene expression related to development, morphogenesis, and tissue repair, indicating potential stem cell-like properties or remodeling functions. These findings collectively highlight the unique and pivotal role of CAFs in facilitating bone metastasis of breast cancer (31).

Functional enrichment and pathway analyses provided further insights into the oncogenic mechanisms of SPOCK1. Genes co-upregulated with SPOCK1 were significantly enriched in several pro-tumorigenic pathways, including the Wnt signaling pathway, PI3K-AKT pathway, TGF-β signaling, and ECM–receptor interaction pathway, all of which are implicated in epithelial–mesenchymal transition (EMT), cell migration, and immune suppression. Moreover, gene set enrichment analysis (GSEA) revealed that the high SPOCK1 expression group was also enriched in inflammation and hypoxia-related pathways, supporting its role in shaping a pro-metastatic and immunosuppressive tumor microenvironment (TME).

Sneha Soni’s research showed that bone marrow-derived mesenchymal stem cells (MSCs) are an important source of CAFs, and can be induced by the TGF-β/SMAD signaling pathway within the breast cancer microenvironment to promote angiogenesis, invasion, and metastasis. Additionally, MSCs can influence EMT processes in breast cancer cells and enhance their metastatic potential. Tumor cells in bone lesions activate osteoclasts, leading to bone matrix degradation and the release of growth factors (e.g., TGF-β, IGF-1), which in turn stimulate tumor growth. Tumor cells subsequently secrete parathyroid hormone-related protein (PTHrP), further activating osteoclasts and establishing a positive feedback loop that promotes bone metastasis (32).

Another study found that CAFs were significantly enriched while immune cells were markedly reduced in breast cancer bone metastases (BoM), compared to primary tumors (PT) and lymph node metastases (LN). The number and heterogeneity of CAFs were notably higher in BoM, suggesting their key role in constructing the metastatic niche and promoting immune suppression. CAFs in BoM actively recruited and remodeled immune cells to establish an immunosuppressive microenvironment through mechanisms such as: recruiting tumor-associated macrophages (TAMs), tumor-associated neutrophils (TANs), and myeloid-derived suppressor cells (MDSCs); promoting regulatory T cells (Tregs) and Th2 polarization; inhibiting cytotoxic T lymphocyte (CTL) infiltration and dendritic cell (DC) function; remodeling ECM and activating focal adhesion kinase (FAK) signaling to block T cell entry into the tumor core; and secreting immunometabolites to suppress immune responses. Key pathways involved include MDK, PTN, SPP1, and FN1, all of which play central roles in immune regulation and tumor progression (33).

In our study, single-cell transcriptomic analysis elucidated the cell type-specific expression pattern of SPOCK1 within the breast cancer microenvironment. We observed that SPOCK1 is primarily expressed in CAFs, with significantly higher expression in tumor-derived CAFs compared to adjacent normal tissues. CAFs are widely recognized as key players in ECM remodeling, angiogenesis, and immune regulation. Our cell–cell communication analysis revealed that SPOCK1-positive CAFs (SPOCK1^+^ CAFs) established stronger interactions with malignant epithelial cells through signaling pathways involving COLLAGEN, FN1, THBS, and IGFBP. These ligand–receptor axes have been extensively implicated in tumor metastasis and tumor–stroma co-evolution.

Strikingly, when stratifying breast cancer by HER2 expression, we found that the communication between SPOCK1^+^ CAFs and epithelial cells was markedly enhanced in HER2-positive subtypes, suggesting that SPOCK1 may contribute to subtype-specific breast cancer progression. SCENIC (34) analysis revealed elevated activity of transcription factors BHLHE41 and MXD4 in SPOCK1^+^ CAFs, indicating a potential transcriptional regulatory network underlying SPOCK1 expression and its downstream effects. Metabolic pathway analysis further demonstrated enhanced activity of glycolysis and sphingolipid metabolism in SPOCK1^+^ CAFs, both of which are known to be closely associated with immune suppression and stromal activation.

Spatial transcriptomics and multiplex immunohistochemistry (mIHC) further validated the spatial co-localization of SPOCK1^+^ CAFs and malignant epithelial cells in the tumor core, reinforcing the spatial-functional link between stromal and tumor cells. These findings suggest that SPOCK1^+^ CAFs may promote tumor cell survival and invasion not only through direct contact and paracrine signaling, but also by establishing an immunosuppressive niche that drives malignant transformation of the TME.

In addition, we investigated the relationship between SPOCK1 and annexin A2 (ANXA2), a known cytoskeletal regulator. The expression of SPOCK1 was significantly positively correlated with ANXA2, implying a potential synergistic role in promoting tumor metastasis. Previous studies have shown that ANXA2 is associated with poor prognosis and metastasis in breast and various other cancers and that ANXA2 enhances osteoclast formation and bone resorption. Our cell communication analysis suggests that SPOCK1^+^ CAFs may regulate ANXA2 expression through an autocrine mechanism involving insulin-like growth factor (IGF) signaling, thereby influencing tumor progression. Ultimately, we found that knockdown of ANXA2 in breast cancer-associated fibroblasts not only affected the expression of SPOCK1 but also led to a decrease in IGF1 expression. Minhua Wu’s study demonstrated that cancer-associated fibroblasts (CAFs) promoted epithelial-mesenchymal transition (EMT) and resistance to epidermal growth factor receptor tyrosine kinase inhibitors (EGFR-TKIs) in non-small cell lung cancer (NSCLC) by secreting hepatocyte growth factor (HGF) and insulin-like growth factor 1 (IGF-1) to activate ANXA2 (35). In addition, several studies have shown that ANXA2 can activate signaling pathways such as PI3K/AKT and transforming growth factor-β (TGF-β), which in turn upregulate the expression of SPOCK1, thereby facilitating EMT, invasion, and metastasis (30). Based on these findings, we hypothesize that in breast cancer, SPOCK1-positive CAFs may involve a synergistic interaction between ANXA2 and SPOCK1 to regulate tumor EMT and metastasis. Moreover, SPOCK1-positive CAFs may further enhance this effect through autocrine signaling via the IGF pathway.

Previous studies have demonstrated a significant increase in both the quantity and heterogeneity of CAFs within bone metastases of breast cancer. Transcriptomic profiling of these CAFs reveals elevated activity in pathways related to tissue repair, development, and morphogenesis, suggesting potential stem-like and remodeling functions. CAFs contribute to a pro-metastatic positive feedback loop by activating osteoclasts, participating in bone matrix degradation, and facilitating growth factor release (36).

As the predominant stromal cell type within the breast cancer tumor microenvironment, CAFs play a critical role in tumor metastasis and progression. They promote epithelial-mesenchymal transition (EMT) of breast cancer cells and enhance their invasive and migratory capabilities through secretion of pro-inflammatory factors such as transforming growth factor-beta (TGF-β), interleukin-6 (IL-6), and interleukin-32 (IL-32), as well as exosomal microRNAs and proteins. Simultaneously, CAFs remodel the extracellular matrix (ECM) and reprogram tumor metabolism, including the reverse Warburg effect, thereby providing both structural support and metabolic sustenance to the tumor. The heterogeneity and dynamic plasticity of CAFs enable them to exhibit distinct functional phenotypes depending on metastatic sites and stages of breast cancer progression, further facilitating immune suppression and therapeutic resistance. Hence, CAFs not only drive breast cancer progression and metastasis but also represent promising therapeutic targets (37).

Siyang Wen et al. reported that CAF-derived IL-32 interacts with integrin β3 on breast cancer cells to activate the p38 MAPK signaling pathway, thereby promoting EMT, invasion, and distant metastasis, ultimately supporting tumor progression. Furthermore, Jiang Ren et al. identified that CAF-secreted Gremlin 1 (Grem1) suppresses the BMP/SMAD signaling axis, enhancing CAF activation and fostering breast cancer cell stemness, invasiveness, and both intravascular and extravascular dissemination, underscoring its critical role in tumor progression (38).

Although the our study provides a relatively preliminary investigation into the intercellular mechanisms compared to previous research and lacks extensive experimental validation, its main objective was to leverage a multi-omics approach to analyze the enrichment of SPOCK1 in cancer-associated fibroblasts and its significant interaction with malignant epithelial cells. This highlights the potential of SPOCK1 as a therapeutic target. Furthermore, *in vitro* experiments exploring the underlying mechanisms of SPOCK1 reinforce the credibility of our findings by providing additional supporting data.

## 5 Conclusion

In summary, our findings highlight SPOCK1 (SPARC/osteonectin, CWCV and Kazal-like domains proteoglycan 1) as a multifunctional key factor broadly involved in tumor biological processes, including immune regulation, extracellular matrix (ECM) remodeling, genomic instability modulation, and intercellular communication. The enrichment of SPOCK1 in cancer-associated fibroblasts (CAFs) and its prominent interactions with malignant epithelial cells underscore its potential as a therapeutic target. Targeting SPOCK1 itself or its downstream signaling axes may disrupt CAF functionality, reduce metastatic potential, and reprogram the tumor immune microenvironment.

Future studies should focus on elucidating the mechanistic roles of SPOCK1 through *in vitro* and *in vivo* functional experiments and further explore its potential for combination therapy in the context of existing immunotherapeutic strategies—particularly in tumors characterized by high CAF abundance and profound immune suppression. Our study underscores the translational relevance of SPOCK1 as a candidate therapeutic target and highlights the unique value of integrating single-cell and spatial transcriptomic approaches to unravel the complexity of tumor–stroma–immune interactions.

## Data Availability

Publicly available datasets were analyzed in this study. This data can be found here: GSE243526 and TCGA.

## Supplementary material

Supplementary Figure 1
**(A)**, Distribution of clusters after single-cell UMAP downscaling; **(B)**, Heatmap of Her2-positive breast cancer tumor cell-associated cellular communication; **(C)**, SPOCK-positive CAF cells self-communicated through the IGF signaling pathway.
