# Supplementary figures and images for: SPOCK1 promotes the progression of breast cancer by modulating cancer-associated fibroblasts and exerts a synergistic effect with ANXA2
Source: Front Oncol. 2025 Aug 5;15:1619171. doi: 10.3389/fonc.2025.1619171 (PMC12361150; doi:10.3389/fonc.2025.1619171)

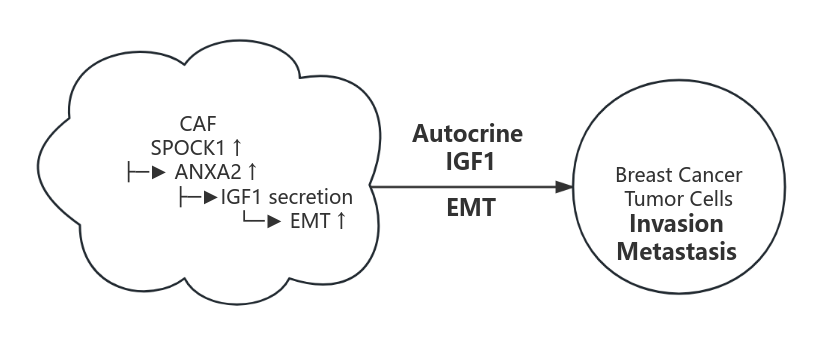

Supplement: Supplementary Figure 1 — (A), Distribution of clusters after single-cell UMAP downscaling; (B), Heatmap of Her2-positive breast cancer tumor cell-associated cellular communication; (C), SPOCK-positive CAF cells self-communicated through the IGF signaling pathway. [file Image1.tif]

Groups CAF-SPOCK1 CAF+SPOCK1

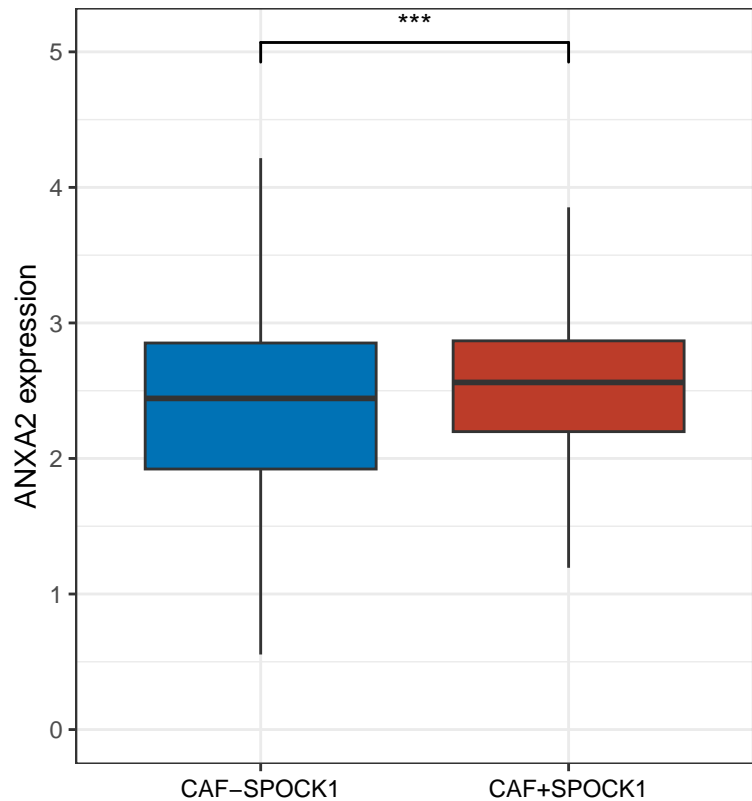

Supplement: Supplementary file 2 [file DataSheet1.pdf]

Groups CAF-SPOCK1 CAF+SPOCK1

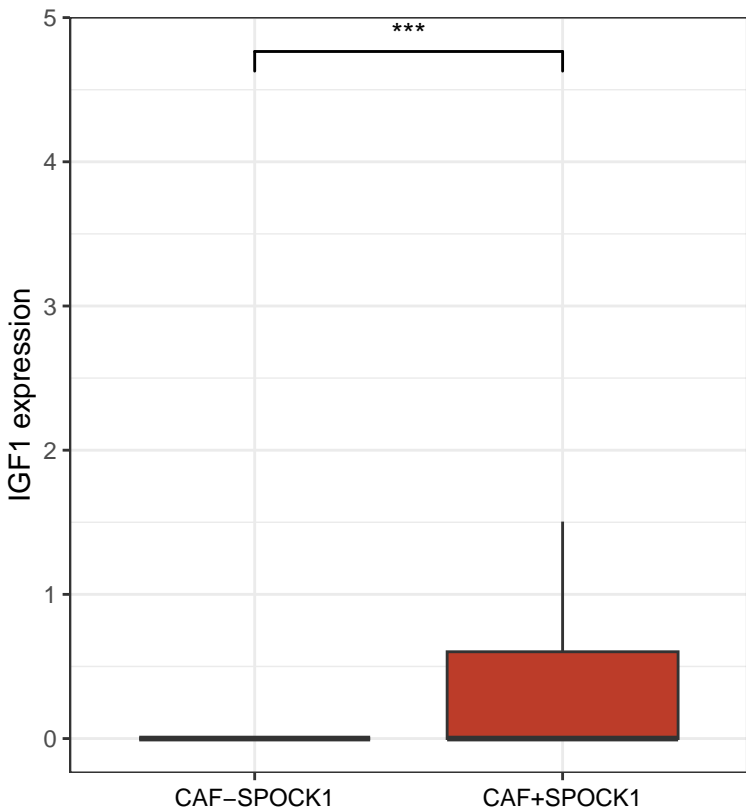

Supplement: Supplementary file 3 [file DataSheet2.pdf]

Groups CAF-SPOCK1 CAF+SPOCK1

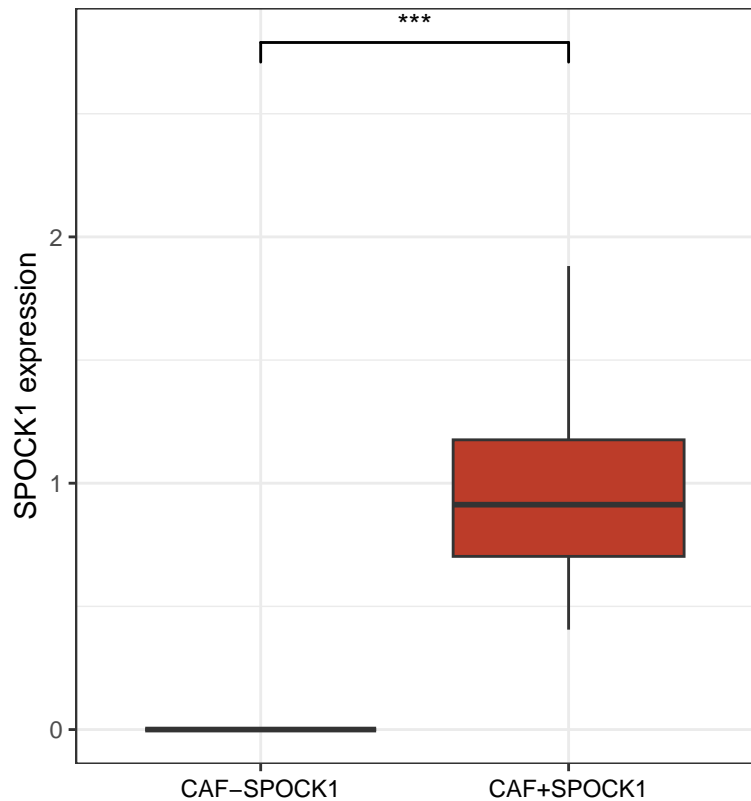

Supplement: Supplementary file 4 [file DataSheet3.pdf]
